# Impact of Ocean Warming and Acidification on Symbiosis Establishment and Gene Expression Profiles in Recruits of Reef Coral *Acropora intermedia*

**DOI:** 10.3389/fmicb.2020.532447

**Published:** 2020-09-30

**Authors:** Youfang Sun, Lei Jiang, Sanqiang Gong, Minglan Guo, Xiangcheng Yuan, Guowei Zhou, Xinming Lei, Yuyang Zhang, Tao Yuan, Jiansheng Lian, Peiyuan Qian, Hui Huang

**Affiliations:** ^1^CAS Key Laboratory of Tropical Marine Bio-resources and Ecology; Guangdong Provincial Key Laboratory of Applied Marine Biology, South China Sea Institute of Oceanology, Chinese Academy of Sciences, Guangzhou, China; ^2^Key Laboratory of Tropical Marine Biotechnology of Hainan Province, Sanya, China; ^3^CAS-HKUST Sanya Joint Laboratory of Marine Science Research and Key Laboratory of Tropical Marine Biotechnology of Hainan Province, Tropical Marine Biological Research Station in Hainan, Chinese Academy of Sciences, Sanya, China; ^4^University of Chinese Academy of Sciences, Beijing, China; ^5^Innovation Academy of South China Sea Ecology and Environmental Engineering, Chinese Academy of Sciences, Guangzhou, China; ^6^Department of Ocean Science and Hong Kong Branch of Southern Marine Science and Engineering Guangdong Laboratory (Guangzhou), Hong Kong University of Science and Technology, Hong Kong, China

**Keywords:** Symbiodiniaceae, symbiosis, juvenile, *Acropora intermedia*, ocean warming, ocean acidification, transcriptomics

## Abstract

The onset of symbiosis and the early development of most broadcast spawning corals play pivotal roles in recruitment success, yet these critical early stages are threatened by multiple stressors. However, molecular mechanisms governing these critical processes under ocean warming and acidification are still poorly understood. The present study investigated the interactive impact of elevated temperature (∼28.0°C and ∼30.5°C) and partial pressure of carbon dioxide (*p*CO_2_) (∼600 and ∼1,200 μatm) on early development and the gene expression patterns in juvenile *Acropora intermedia* over 33 days. The results showed that coral survival was >89% and was unaffected by high temperature, *p*CO_2_, or the combined treatment. Notably, high temperature completely arrested successful symbiosis establishment and the budding process, whereas acidification had a negligible effect. Moreover, there was a positive exponential relationship between symbiosis establishment and budding rates (*y* = 0.0004e^6.43x^, *R* = 0.72, *P* < 0.0001), which indicated the importance of symbiosis in fueling asexual budding. Compared with corals at the control temperature (28°C), those under elevated temperature preferentially harbored *Durusdinium* spp., despite unsuccessful symbiosis establishment. In addition, compared to the control, 351 and 153 differentially expressed genes were detected in the symbiont and coral host in response to experimental conditions, respectively. In coral host, some genes involved in nutrient transportation and tissue fluorescence were affected by high temperature. In the symbionts, a suite of genes related to cell growth, ribosomal proteins, photosynthesis, and energy production was downregulated under high temperatures, which may have severely hampered successful cell proliferation of the endosymbionts and explains the failure of symbiosis establishment. Therefore, our results suggest that the responses of symbionts to future ocean conditions could play a vital role in shaping successful symbiosis in juvenile coral.

## Introduction

The obligate symbiosis between scleractinian corals and symbionts (family Symbiodiniaceae) is fundamental to the high-level production of reef corals and coral reefs in the oligotrophic tropical oceans. Over the past few decades, however, symbiosis has been affected by many environmental stressors, which can cause coral bleaching and even mortality (Hughes et al., 2017; Van Oppen and Lough, 2018). The main triggers for bleaching events are elevated sea surface temperature (SST) and ocean acidification (Anthony et al., 2008; Hughes et al., 2017). While ocean acidification and warming are known to decrease skeleton development and cause bleaching in adult corals (Foster et al., 2015), their combined effects on juvenile corals are less understood. Juvenile corals have been shown to be extremely sensitive to environmental stress than adults (Fabricius, 2005; Byrne and Przeslawski, 2013). The persistence of coral reefs is partially dependent on the successful dispersal and recruitment of early life phases of coral (Harrison, 2011). Based on the Intergovernmental Panel on Climate Change (IPCC, 2007) report, the SST is expected to increase by 2–3°C, and surface ocean pH is expected to decline by ∼0.3 by 2100; this will result in a significant threat to juveniles, especially in symbiosis (Hughes et al., 2003; Cziesielski et al., 2019).

The relationship between the stony coral host and Symbiodiniaceae, including systematic identification and symbiont transmission, has been extensively studied. Seven genera (previously nine deeply divergent clades) within Symbiodiniaceae have been formally described based on their genetics and ecology: *Symbiodinium* (Clade A), *Breviolum* (Clade B), *Cladocopium* (Clade C), *Durusdinium* (Clade D), *Effrenium* (Clade E), *Fugacium* (Clade F), and *Gerakladium* (Clade G). Each genus includes several formally described molecular types (Lajeunesse et al., 2018). Moreover, the majority of reef-building corals (∼80%) establish symbiosis relationships with algal symbionts *via* horizontal transmission (Baird et al., 2009), acquiring symbionts at larval or juvenile stages that are the most suitable for the surrounding settlement environment (Schwarz et al., 1999). Despite the significance of the early life stages to overall coral reproduction, recruitment, and recovery, as well as the sensitivity of these life stages to environmental change, recent studies have primarily focused on the adaptation and response of adult corals to climate change (Silverstein et al., 2015; Davies et al., 2018). Specifically, the role of increased temperature and partial pressure of carbon dioxide (*p*CO_2_) on symbiosis establishment in the juvenile stage has not been fully addressed.

It has been found that juvenile and larval corals may possess the ability to select and alter symbiont types and abundance in response to climate change. For example, juvenile *Acropora tenuis* and *Acropora millepora* preferentially select *Durusdinium*, and the relative abundance of *Durusdinium* spp. increased is high in *A. tenuis* juveniles under elevated temperature conditions (Abrego et al., 2012; Yorifuji et al., 2017). Similarly, Schnitzler et al. (2012) found that high temperature inhibits the ability of aposymbiotic *Fungia scutaria* larvae from establishing a symbiosis with *Cladocopium* spp. Overall, these studies suggest that corals harboring *Durusdinium* spp. may exhibit high thermal tolerance. Moreover, the acquisition of symbionts by *Acropora digitifera* juveniles decreased with an increase in seawater acidity (Suwa et al., 2010). Abrego et al. (2008) suggested that *A. tenuis* juveniles associated with *Cladocopium* spp. exhibit higher thermal tolerance than those associated with *Durusdinium* spp., indicating that the relationship between coral endosymbionts and the thermal tolerance of juvenile corals is not fully understood.

In this study, to develop a better understanding of the establishment of symbiosis and driving mechanisms during the onset of juvenile coral–algae symbiosis under climate change, we investigated the effect of elevated temperature (∼28.0°C and ∼30.5°C) and acidification (pH ∼8.12 and ∼7.85) for 33 days on the establishment of symbiosis in juvenile *A. intermedia*. We also measured the physiological parameters of survival, budding, symbiosis establishment, and green fluorescence in juvenile *A. intermedia*. Gene expression patterns of multiple genes in both coral host and symbionts are representatives of key cellular processes (e.g., energy metabolism and protein synthesis) occurring under different experimental conditions. Our results show the responses of juvenile *A. intermedia* to possible future thermal and *p*CO_2_ stressors and provide valuable information on the onset of symbiosis and its underlying mechanisms. Furthermore, investigating the effects of elevated temperature and acidification on the onset of symbiosis in juvenile corals will provide new insights into the biological and ecological consequences of global climate change on scleractinian corals.

## Materials and Methods

### Coral Collection and Larval Culture

Five colonies of *A. intermedia* were collected on April 18, 2017, from the Luhuitou fringing reef (18°12′N, 109°28′E) in Sanya, Hainan Island, China, with the permission of the Administration of the Sanya Coral Reef National Nature Reserve. The coral samples were immediately transferred to the Tropical Marine Biological Research Station in Hainan and placed in outdoor aquaria with flow-through seawater supply. Gametes were collected from different coral colonies between 21:00 and 21:30 h. Five days post-fertilization (20:00 h, April 23), ∼50–60 larvae were transferred to each Petri dish containing autoclaved chips of *Porolithon onkodes*, which is recognized as the settlement substrate for *Acropora* coral larvae (Heyward and Negri, 1999). We allowed the larvae to settle for 24 h and subsequently removed overlapping settlements and non-target recruits, leaving ∼20 recruits in each Petri dish. Additionally, three *A. intermedia* nubbins from different colonies were preserved in 90% alcohol for downstream symbiont analysis.

### Juvenile Cultures Under Experimental Treatments

Newly settled recruits were then cultured under four different treatments with three replicate tanks per treatment: ATAC as the control [ambient temperature (∼28°C; the average seawater temperature during the coral spawning season in this region) and ambient *p*CO_2_ (∼600 μatm; ∼8.12 pH)], ATHC [ambient temperature and high *p*CO_2_ (∼1,200 μatm; ∼7.85 pH)], HTAC [high temperature and ambient *p*CO_2_ (∼600 μatm; ∼30.5°C)], and HTHC [high temperature and high *p*CO_2_ (∼1,200 μatm; ∼30.5°C)]. The treatments were designed based on the predicted oceanic temperatures and pH levels for 2100 (IPCC, 2007).

Specifically, three to four Petri dishes containing the settled recruits were placed in each flow-through tank (3 L h^–1^) containing 20 L of sand-filtered seawater pumped at a depth of ∼5 m from the nearby reef area. The seawater was mixed and pumped at a rate of 600 L h^–1^ with a submerged pump (Weipro, China), and the seawater pH was manipulated following a previously described protocol (Jiang et al., 2017). The seawater pH was manipulated by pH controllers (accuracy: ±0.1 pH, Weipro, China) equipped with solenoid valves to regulate the injection of pure CO_2_ and maintain the target pH. In contrast, the control treatment received a supply of outside air. The seawater temperature was maintained using a heater (Weipro, China) (accuracy: ±1°C). The temperature was monitored at 30-min intervals using a HOBO Pendant, temperature data logger (Onset, Bourne, MA, United States). Lastly, the tanks were maintained under a cycle of 12 h darkness and 12 h light (light intensity ∼200 μmol photons m^–2^ s^–1^), and the light intensity was ∼200 μmol photons m^–2^ s^–1^. During the 33-day experiment, the recruits were checked daily, and the tanks and tiles were cleaned every 4 days to remove turf algae.

At 5-day intervals, 50 ml seawater samples were collected from each tank and preserved in saturated HgCl_2_ for the determination of total alkalinity (TA) with an automatic Total Alkalinity Titrator (AS-ALK2, Apollo, United States). Temperature, salinity, and pH were monitored and adjusted twice daily with a pH meter (SevenGo, Mettler Toledo, Switzerland). The pH probe was calibrated every other day utilizing a two-point calibration technique with 7.00 and 10.00 pH buffers. The carbonate parameters were calculated using CO2SYS (Lewis et al., 1998). The seawater conditions for each treatment are shown in Table 1.

**TABLE 1:** Experimental seawater conditions for each treatment.

**Treatment**	**pH_T_**	**Temperature (°C)**	**Salinity**	**TA (μmol kg^–1^)**	***p*CO_2_ (μatm)**	**Ω_Arag_**
ATAC	8.12 ± 0.008	28.0 ± 0.008	33.43 ± 0.05	2,327 ± 40.04	581.85 ± 41.59	3.17 ± 0.24
ATHC	7.86 ± 0.006	27.98 ± 0.012	33.44 ± 0.58	2,279 ± 29.00	1,174.84 ± 45.85	1.75 ± 0.06
HTAC	8.12 ± 0.008	30.5 ± 0.006	33.46 ± 0.05	2,279 ± 31.52	608.51 ± 53.45	3.25 ± 0.24
HTHC	7.85 ± 0.007	30.4 ± 0.005	33.60 ± 0.05	2,328 ± 34.89	1,221 ± 38.49	1.91 ± 0.06

*Carbonate chemistry parameters: pH_T_, pH total scale; TA, temperature, salinity, total alkalinity; *p*CO_2_, partial pressure of carbon dioxide; Ω_Arag_, aragonite saturation. Treatment conditions were as follows: ATAC (28°C and *p*CO_2_ ∼600 μatm), ATHC (28°C and *p*CO_2_ ∼1,200 μatm), HTAC (30.5°C and *p*CO_2_ ∼600 μatm), HTHC (30.5°C and *p*CO_2_ ∼1,200 μatm).*

At the end of the experiment, recruits in each Petri dish were checked under a dissecting microscope (SZX7, Olympus, Japan) and scored as alive or dead based on the presence of typical translucent tissue (Abrego et al., 2008). Survivorship of recruits was calculated for each replicate dish as the number of living recruits divided by the initial number of recruits. In addition, the symbiotic status of recruits was defined as follows: (1) aposymbiotic or low abundance of symbionts when the polyp tissue was translucent and lacked pigmentation, with the white skeleton clearly visible under the tissue, and (2) symbiotic when symbiont cells were clearly present and when the oral disk and tentacles of juvenile polyps were brown and pigmented (Abrego et al., 2012). It is important to note that although this visual assessment of symbiont uptake success could not preclude the possibility of symbiont infection, it did represent a good proxy for the establishment of symbiosis, where symbiont cells had not only entered the coral cells but also reproduced successfully *in hospite*; hereafter referred to as symbiosis establishment (Abrego et al., 2012; Nitschke et al., 2016; Ali et al., 2019). The symbiosis establishment rates were calculated as the proportion of pigmented juveniles relative to the total number of living recruits on the last day, and the budding state of each recruit was determined by recording the occurrence of new buds on the periphery of primary polyps. In addition, using a dissecting microscope, photographs of the polyps were analyzed to determine the budding rates (Humanes et al., 2016).

Furthermore, we found that the tissue of many recruits in the high-temperature treatments exhibited bleaching-induced fluorescence, and therefore, we also determined the percentage of this type of recruit using a dissecting microscope (Roth and Deheyn, 2013). At each census, the number of living recruits was recorded for each treatment. The remaining coral juveniles were preserved in liquid nitrogen and stored at −80°C for transcriptome analysis.

Statistical analyses were conducted in IBM SPSS. The effects of temperature and *p*CO_2_ on survival, budding, the onset of symbiosis, and green fluorescence were analyzed using a generalized linear model.

### Transcriptome Sequencing

Total RNA of each coral juvenile sample was extracted using a TRIzol^®^ Reagent RNA Isolation Kit (Invitrogen, Grand Island, NY, United States) following the manufacturer’s instructions. After a quality check, 12 RNA samples were stored at −80°C and transferred to Novogene Biological Information Technology Co., Ltd. (Beijing, China), for paired-end sequencing. All 12 libraries were sequenced on the Illumina HiSeq X Ten platform. Clean data were obtained by removing sequences containing adapters, reads containing poly-N, and low-quality reads from the raw data. In addition, we calculated the base quality scores (Q20 and Q30) and determined the GC (guanine-cytosine)-content (Supplementary Table 1).

### Transcriptome Assembly

Transcriptomes were assembled *de novo* based on the left.fq and right.fq files from all libraries using the Trinity program (Grabherr et al., 2011) with the default setting. A total of 103,285 unigenes were obtained. To identify the unigenes that were derived from either the coral host or the symbiont, we used BLASTN (coverage > 95%, identity > 90%, E < 1E^–6^) to map the database containing cnidarian and Symbiodiniaceae genomes and transcriptomes (Supplementary Table 2). Unigenes were mapped against database containing cnidarian genome and transcriptome using BLASTN, and the obtained sequences were called coral-all unigenes. The coral-all unigenes that could not be mapped to the Symbiodiniaceae database using BLASTN were considered coral unigenes and subsequently named as coral-specific unigenes. In addition, the unigenes were mapped against the database containing Symbiodiniaceae genome and transcriptome using BLASTN (coverage > 95%, identity > 90%, E < 1E^–6^), and the obtained sequences were called symbio-all unigenes. The symbio-all unigenes that could not be mapped to the cnidarian database using BLASTN were considered Symbiodiniaceae unigenes and subsequently named symbio-specific unigenes. The coral-specific and symbio-specific unigenes were used for downstream analysis.

### Differential Expression Analysis

The reads per kilobase per million mapped reads (RPKM) value was used to assess unigene expression levels using RESM software (Mortazavi et al., 2008). The RPKM calculation formula is as follows: RPKM = (1,000,000 × C)/(N × L/1,000). Given RPKM (A) to be the expression of unigene A, C is the number of reads that are uniquely mapped to unigene A, N is the total number of reads that are uniquely mapped to all unigenes, and L is the length of unigene A. The RPKM method can eliminate the influence of different gene lengths and amount of sequencing data in the calculation of unigene expression. Additionally, unigenes were annotated by sequence similarity search using public databases: the Swiss-Prot, NCBI Non-redundant protein (Nr), and KOG databases using BLASTX, BLASTN, and BLASTP, respectively, with *E* value < 10^–5^. In addition, three comparisons were made to examine the changes in coral and symbiont gene expression between the treatments ATHC, HTAC, and HTHC and the control (ATAC): ATHC treatment vs. control; HTAC treatment vs. control, and HTHC treatment vs. control. Subsequently, we utilized the R package, DESeq2, to identify differentially expressed genes (DEGs) (Mohamed et al., 2019) with thresholds of log_2_ (foldchange) > 2 and a false discovery rate (FDR) < 0.01. Using a model based on the negative binomial distribution, DESeq2 provided statistical routines for determining differential expression in the digital gene expression data.

### Identification of Symbiodiniaceae

To identify the Symbiodiniaceae genera present in juvenile *A. intermedia*, we conducted a local BLAST search of the symbiont transcriptome against a database containing chloroplast 23S rDNA and nuclear 28S rDNA sequences (Pochon et al., 2006). In contrast, to identify the Symbiodiniaceae genera present in adult *A. intermedia*, we performed high-throughput sequencing of the internal transcribed spacer 2 (ITS2) region of ribosomal RNA gene amplicons, following the protocols described by Zhou et al. (2017). Total DNA was extracted as described previously (Zhou et al., 2012). The purity of the DNA was measured using a NanoDrop spectrophotometer (Thermo Fisher Scientific, United States). The DNA samples were stored at −20°C for further analysis. For library preparation, the DNA samples were amplified using polymerase chain reaction (PCR) primers employed by Lajeunesse and Trench (2000). The ITS2 amplicon was approximately 320 base pairs long. The primer sequences were 5′- AGATCGGAAGAGCGTCGTGTAGGGAAAGAGTGT N_6_ GAA TTGCAGAACTCCGTG-3′ (ITSintfor2) and 5′-AGATCGGA AGAGCACACGTCTGAACTCCAGTCACN_6_ GGGATCCATAT GCTTAAGTTCAGCGGGT-3′ (ITS2-reverse), targeting the ITS2 region of the ribosomal RNA gene for Symbiodiniaceae. Primers included Illumina library adapters (underlined) and a bar code (shown as N). Then, PCR was performed with 12.5 μl of PCR reagent (Bio-Rad, United States), 0.1 μM primer, 50 ng of DNA, and DNase-free water to make a total volume of 25 μl. The following procedure was implemented: initial denaturation for 3 min at 94°C, followed by 34 cycles at 98°C for 10 s, 51°C for 30 s, 68°C for 30 s, and a final extension step of 5 min at 68°C. The PCR products were validated using an Agilent 2100 Bioanalyzer (Agilent Technologies, Palo Alto, CA, United States) and quantified with a NanoDrop spectrophotometer. All qualified amplification products were mixed in equal amounts followed by sequencing on an Illumina MiSeq instrument (Illumina, San Diego, CA, United States) according to the manufacturer’s instructions using a 300 × 2 paired-end configuration. The raw data were submitted to the National Center for Biotechnology Information (NCBI) Sequence Read Archive (PRJNA633301). Processing and analysis of the ITS2 sequences were conducted following our previously described protocol (Gong et al., 2018).

## Results

### Response of Coral Juveniles to Elevated Temperature and Partial Pressure of Carbon Dioxide

**FIGURE 1:**
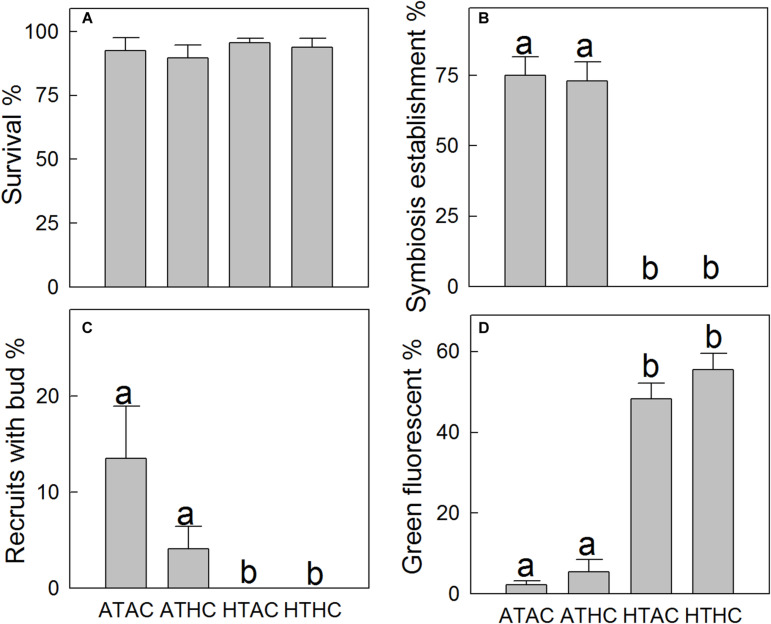
Mean (±SE) survival (**A**, *n* = 32), symbiosis establishment (**B**, *n* = 32), budding (**C**, *n* = 32), and green fluorescence (**D**, *n* = 32) of juvenile *Acropora intermedia* under the four treatments after 33 days. Treatment conditions were as follows: ATAC (28°C and pCO_2_ ∼600 μatm), ATHC (28°C and *p*CO_2_ ∼1,200 μatm), HTAC (30.5°C and *p*CO_2_ ∼600 μatm), HTHC (30.5°C and *p*CO_2_ ∼1,200 μatm). The significance of difference within pairs of groups was assessed using the Bonferroni test (*P* < 0.05, different letters indicate statistically significant differences).

**FIGURE 2:**
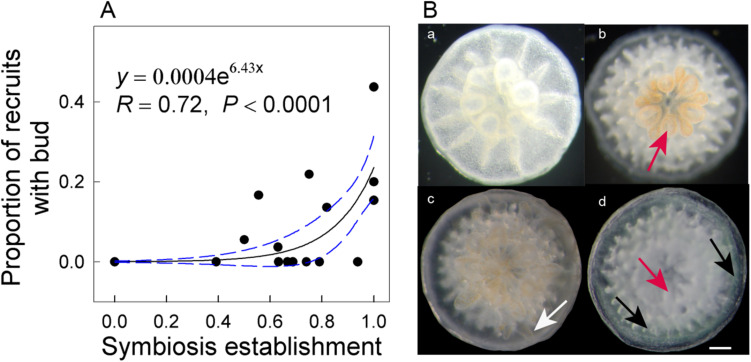
Exponential relationship between symbiosis establishment and budding rates of *Acropora intermedia* after 33 days **(A)**. Developmental stages of *A. intermedia* after settlement **(B)**. **(a)** One-day-old aposymbiotic polyp, **(b)** 7-day-old polyp with visible symbionts within oral disk and tentacles, **(c)** 28-day-old small colony with a new bud (white arrow) in the periphery area, **(d)** 28-day-old recruit with normal translucent (red arrow) and green fluorescent tissues (black arrows). Scale bar = 1 mm. Note: each data point represents an individual Petri dish.

Higher temperatures arrested symbiosis establishment (*F*_1,28_ = 248.11, *P* < 0.001) and reduced budding in *A. intermedia* juveniles (*F*_1,28_ = 8.868, *P* = 0.006), while the effects of reduction in pH were not significant (*F*_2,28_ = 2.533, *P* = 0.123). The mean symbiosis establishment rates for ambient temperature treatments were ∼70% but 0 for high-temperature treatments (Figure 1B). In addition, 13.48% and 4.09% of recruits successfully budded under the ATAC and ATHC treatments, respectively. In contrast, no recruits budded successfully under higher temperature treatments (Figure 1C). Moreover, at the end of the 33-day experimental period, an exponential relationship (*y* = 0.0004e^6.43x^, *R* = 0.72, *P* < 0.0001) existed between symbiosis establishment and budding rates (Figure 2A). All the data points in Figure 2A are from individual Petri dishes of the four treatments. The aposymbiotic polyp, symbiosis establishment, and budding are shown in Figures 2Ba–c, respectively.

The survival rates of coral recruits ranged from ∼89 to 96% across all treatments (Figure 1A), and the statistical results (Supplementary Table 3) show that the survival rates did not differ significantly between treatments. However, higher temperature significantly elevated green fluorescence in tissues (*F*_1,28_ = 217.715, *P* < 0.001). The percentage of tissue expressing green fluorescence under ambient temperature treatments was ∼4%, but under high-temperature treatments, it was ∼50% (Figures 1D, 2B,d). At high temperatures, the number of corals exhibiting vibrantly green fluorescence increased 10-fold.

### Assembly of the Reference Transcriptome

The obtained coral holobiont transcriptome was 88.19 Mb with an N50 size of 1,522 bp. The maximum, minimum, and mean lengths of the unigenes were 21,321,201, and 853 bp, respectively. The GC-content was 41.60%. The N50 of the coral transcriptome was 1,085 bp, with a mean unigene length of 697 bp, and a GC-content of 39.90%. Lastly, the N50 of the symbiont transcriptome was 472 bp, with a mean unigene length of 434 bp and a GC-content of 57.11% (Supplementary Table 4). The raw data have been submitted to the NCBI under accession numbers SRR10305223–SRR10305234.

### Differential Gene Expression

Our analyses revealed 154 DEGs within the coral host. Specifically, under the ATHC, HTAC, and HTHC treatments, 3, 77, and 74 genes, respectively, were differentially expressed in the coral host. For the symbiont, 351 genes were differentially expressed (0, 133, and 218 under the ATHC, HTAC, and HTHC treatments, respectively) (Supplementary Figure 1). Although the number of DEGs increased under HTAC and HTHC treatments, the number of downregulated DEGs far exceeded that of upregulated ones.

### Differentially Expressed Gene Functions

**FIGURE 3:**
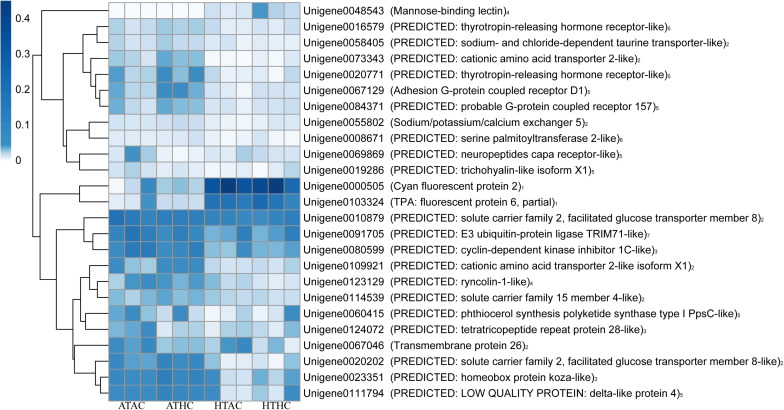
Heatmap of annotated unigenes in the coral host in the ATAC (28°C and *p*CO_2_ ∼600 μatm), ATHC (28°C and *p*CO_2_ ∼1,200 μatm), HTAC (30.5°C and *p*CO_2_ ∼600 μatm), and HTHC (30.5°C and *p*CO_2_ ∼1,200 μatm) treatments. Note: numbers 1–7 represent fluorescent proteins, transport protein, cell proliferation, immunity-related factors, signal transduction, lipid or fatty acid metabolism, and stress category, respectively. The heatmap was drawn based on the log-transformed RPKM value of unigenes. The color bar in the heatmap represents the variation of the value of RPKM (from white to blue means the expression value of unigene from 0 to 0.4). High expression (blue color) represents the high value of RPKM, which means that the expression level of unigene among different groups or different unigenes is high. Unigenes were functionally categorized based on database searches and literature.

**FIGURE 4:**
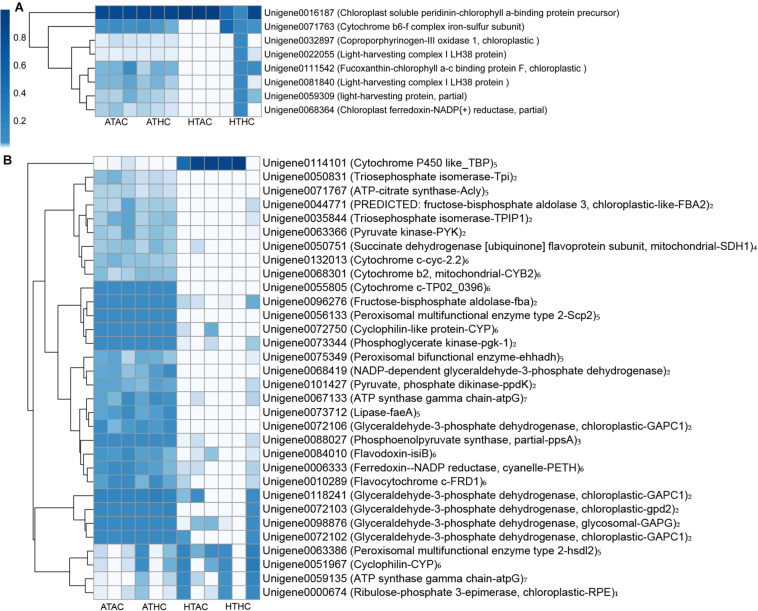
Heatmap of annotated unigenes related to photosynthesis **(A)** and metabolic processes **(B)** in the symbionts under the ATAC (28°C and *p*CO_2_ ∼600 μatm), ATHC (28°C and *p*CO_2_ ∼1,200 μatm), HTAC (30.5°C and *p*CO_2_ ∼600 μatm), and HTHC (30.5°C and *p*CO_2_ ∼1,200 μatm) treatments. Number 1–7 represents Calvin cycle, glycolysis, gluconeogenesis, TCA cycle, lipid or fatty acid metabolism, respiration chain and ATP synthesis category, respectively. The heatmap was drawn based on the log-transformed RPKM value of unigenes. The color bar in the heatmap represents the variation of the value of RPKM (from white to blue means the expression value of unigene from 0 to 1). High expression (blue color) represents the high value of RPKM, which means that the expression level of unigene among different groups or different unigenes is high. Unigenes were functionally categorized based on database searches and literature.

Unigenes were categorized based on functional similarity, while the search processes were based on sequence similarity. Unigenes were annotated by BLASTX against the Swiss-Prot, BLASTN against the Nr, and BLASTP against the KOG databases with *E* value < 10^–5^. The function of unigenes was grouped according to the public database search and literature (Desalvo et al., 2008). As a result, the functional categories of stress, transport protein, lipid or fatty acid metabolism, cell proliferation, fluorescent proteins, signaling, transcription factors, cell immune response, and others were related to the coral host (Figure 3 and Supplementary Figure 2). Using the same method, the DEG functions of the symbionts were also classified into 16 functional groups: stress, photosynthesis, Calvin cycle, glycolysis, gluconeogenesis, tricarboxylic acid (TCA) cycle, lipid or fatty acid metabolism, respiratory chain, protein metabolism, amino acid and vitamin metabolism, DNA replication, transcription, proton ion protein transportation, transport protein, flagellum-related factors, and others (Figure 4 and Supplementary Figure 2).

### Identification of Symbiodiniaceae Communities

**FIGURE 5:**
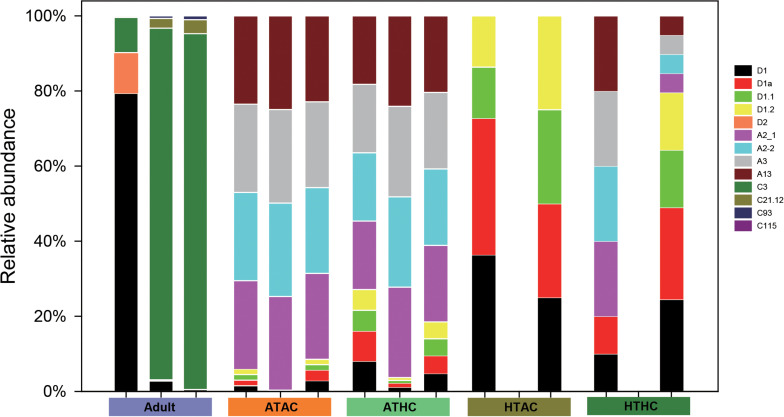
Symbiodiniaceae diversity in adult and juvenile *Acropora intermedia*. Adult refers to adult *A. intermedia*. ATAC, ATHC, HTAC, and HTHC refer to juvenile coral in four treatments: ATAC (28°C and pCO_2_ ∼600 μatm), ATHC (28°C and pCO_2_ ∼1,200 μatm), HTAC (30.5°C and pCO_2_ ∼600 μatm), and HTHC (30.5°C and pCO_2_ ∼1,200 μatm). Three replicates per treatment. Symbionts were not detected in one replicate of HTAC and HTHC treatments.

In juvenile *A. intermedia*, there were significant differences in the symbiont communities between treatments, suggesting that coral–algal symbiosis is highly flexible. Notably, these differences were attributed to changes in the relative abundance of existing *Durusdinium* spp. under high temperatures (Figure 5). The Symbiodiniaceae associated with the ATAC and ATHC treatments were mainly *Symbiodinium* spp. Of three replicates of the HTAC treatment, two were occupied by *Durusdinium* spp., whereas the other was not detected. At the subclade level, D1, D1a and D1.1, and D1.2 dominated in the HTAC treatment. Of three replicates of the HTHC treatment, two were occupied by both *Durusdinium* spp. and *Symbiodinium* spp., while the other was not detected. At the subclade level, the abundance of each of D1, D1a, A2-1, A2-2, and A3 was higher. In contrast, C3 and D1 were the most abundant Symbiodiniaceae species in adult *A. intermedia* (Figure 5).

## Discussion

Understanding the effects of global climate change on coral requires information not only concerning its the direct impact on physiological indicators but also on gene expression levels. In this study, physiological responses and a large number of differentially expressed transcripts were observed in juvenile corals in the experimental conditions. Firstly, all aposymbiotic juveniles in the four treatments were of the same status before culturing. Secondly, at the end of the 33-day experiment, the juveniles had already shown different responses (e.g., symbiont community). As a result, all the different responses are likely attributable to the experimental conditions.

Ocean warming might be more deleterious for symbiosis establishment than acidification. Elevated temperature may not favor symbiosis onset, and this could affect asexual budding reproduction. Temperature stress reduces ribosomal protein gene expression in the symbionts and upregulates fluorescent protein gene expression in the coral host. This study yielded novel evidence of symbiont reproduction and coral host stress response under heat stress conditions. The results may provide a more comprehensive understanding of the influences of elevated temperature and *p*CO_2_ on symbiosis establishment in juvenile corals.

### Response of Symbiodiniaceae to Elevated Temperature

Although elevated temperature impeded the onset of symbiosis, there are symbionts in the juvenile corals based on the RNA-seq data under ATAC and ATHC treatments, suggesting that the method of using a dissecting microscope to detect the symbiont infection may not be very accurate. Under ambient temperature conditions, the main components of the Symbiodiniaceae found in the recruits were *Symbiodinium* spp., but in response to thermal stress, *Durusdinium* spp. became predominant, indicating that juveniles are likely to become locally adapted through shifts in symbiont community composition. Juveniles possibly adapt to a high temperature by forming novel host–Symbiodiniaceae combinations and taking on symbionts that can tolerate warm conditions. Mounting evidence suggests that scleractinian corals may be able to choose algal symbiont types in response to increasing seawater temperatures (Abrego et al., 2009; Yorifuji et al., 2017) and ocean acidification (Tong et al., 2017). For adult corals, the dominant symbionts were *Cladocopium* and *Durusdinium* spp. Distinct symbiont communities were detected in juvenile and adult *A. intermedia*, suggesting that resilience to thermal stress is associated with a highly flexible symbiotic association.

Most of the downregulated genes identified in the symbionts were related to the encoding for ribosomal proteins (Supplementary Figure 3). Ribosomal proteins of *Montastraea faveolata* are downregulated in response to heat shock (Voolstra et al., 2009; Polato et al., 2010). In contrast, Kenkel et al. (2017) suggested that the upregulation of ribosomal proteins in symbionts resulted in increased cell growth and, thus, elevated net photosynthesis. Ribosomal function is associated with protein synthesis and, thus, cell growth through regulation of the cell cycle and cell size (Jorgensen and Tyers, 2004). Moreover, under high-temperature conditions, DNA replication-related genes were downregulated, probably further contributing to reduced symbiont cell growth and reproduction. Thus, the reduction in symbiont cell growth and development may have inhibited the successful establishment of symbiosis under high temperatures.

The transcriptome data revealed that the carbohydrate metabolism of the symbionts decreased because of high temperature. Some genes related to enzymes of photosynthesis, glycolysis, and TCA cycle were downregulated under high-temperature treatments (Figure 4). Regarding photosynthesis, when symbionts are exposed to elevated temperatures, some critical proteins (e.g., light-harvesting protein and fucoxanthin–chlorophyll a–c binding protein F) are damaged, and this may result in the dysfunction of photosystem II (Warner et al., 1999). Still, heat stress may damage the thylakoid membranes, causing them to less produce ATP and nicotinamide adenine dinucleotide phosphate (NADPH) (Weis, 2008). Therefore, this result suggests that prolonged exposure to heat stress may cause symbiont photoinhibition, resulting in reduced carbon translocation from the symbiont to the coral host (Hillyer et al., 2017b). Moreover, pyruvate kinase (limited enzyme in glycolysis) is downregulated under high temperatures, less producing pyruvate ATP (Fernie et al., 2004). Pyruvate is then fed into the TCA cycle to generate less ATP. Regarding the reduction of glycolysis and photoinhibition, similar effects on symbionts are produced by marine pollutants such as 1,3,5-trinitro-1,3,5 triazine (Gust et al., 2014). A gene (unigene0050751) that encoded an essential enzyme [succinate dehydrogenase (ubiquinone) flavoprotein subunit, mitochondrial] within the TCA cycle was significantly downregulated [log_2_(foldchange) = −20.09] in the symbiont transcriptome. Therefore, there was a probable reduction in ATP production given that symbiont photoinhibition has been previously reported to be the result of a reduction in symbiont carbohydrate metabolism (Lesser, 1997).

Lastly, regarding the symbiont DEGs, it has been confirmed that the differentially expressed transcripts of *C. goreaui* and *D. trenchii* in juveniles were different (Yuyama et al., 2018). However, the determination of experimental conditions or symbiont species that account for these DEGs requires further investigation, since it cannot be addressed from the data obtained in the study – as in a previous study (Davies et al., 2018).

### Survival, Symbiosis Establishment, and Asexual Budding

Our finding that the survivorship of juvenile *A. intermedia* remained ∼96% after 33-day exposure to high temperature is in agreement with results of Foster et al. (2015), who found no significant effect of elevated temperature on the post-settlement survival rate of juvenile *A. spicifera*. In the present study, the most plausible explanation for the lack of an elevated temperature effect on survivorship is unsuccessful symbiont infection under thermal stress. Yakovleva et al. (2009) suggested that symbionts can cause an increase in oxidative stress in coral larvae under heat stress, resulting in increased reactive oxygen species (ROS) and tissue damage, leading to reduced larval survival. Therefore, juvenile corals under high temperatures would be more resistant to temperature stress due to lower ROS production, thus favoring their survival. However, budding and symbiosis onset were significantly affected by high temperature. Budding is likely to be an energetically expensive process for newly settled coral and potentially strongly dependent on the successful establishment of symbiosis (Graham et al., 2013). Indeed, budding and symbiosis establishment rates formed a linear relationship (*y* = 0.0004e^6.43x^, *R* = 0.72, *P* < 0.0001) in *A. intermedia*, possibly indicating that symbiosis establishment is positively correlated with budding in the coral host and that budding potentially benefits from symbiosis establishment.

### Response of Fluorescent Protein Genes in Coral Host to Elevated Temperature

In this study, there was an increase in green fluorescent tissue in juvenile coral, and two genes (unigene103324 and 0000505) encoding fluorescent proteins (e.g., TPA: fluorescent protein 6, partial, and cyan fluorescent protein 2) were indeed upregulated 4.5–7.7-fold under elevated temperature conditions. Similarly, Yuyama et al. (2012) revealed that juvenile *A. tenuis* dominated by *Durusdinium* spp. highly expressed green fluorescent proteins (GFPs) under heat stress. Fluorescent proteins in corals exhibit ROS-scavenging ability and are generally regarded as one of the host-driven protective mechanisms that could contribute to the regulation of the bleaching response (Palmer et al., 2009; Bollati et al., 2020). The phenomenon of green fluorescence is highly expressed in response to high temperature in adult corals owing to a family of GFP-like proteins and is called colorful bleaching (Bollati et al., 2020). Thus, the overexpression of fluorescent proteins in coral that hosted *Durusdinium* spp. may enhance resistance and persistence under heat stress.

### Response of Transport Protein Genes in Coral Host to Elevated Temperature

Several sets of genes encoding transport protein were downregulated in high temperatures, such as *Tret1*, *SLC7A1*, and *SLC7A2*. *Tret1* codes for a “PREDICTED: solute carrier family 2, facilitated glucose transporter member 8-like” protein, while *SLC7A1* and *SLC7A2* code for “PREDICTED: cationic amino acid transporter 2-like isoform X1” and “PREDICTED: cationic amino acid transporter 2-like isoform,” respectively. These two amino acid transporters translocate L-arginine produced by symbiont algae from the symbiont to the coral host (Ferrier, 1994; Cérec et al., 2007), although glucose is the major photosynthetic product translocated from symbionts to coral host (Hillyer et al., 2017a). The onset of symbiosis was not successfully established under heat stress, suggesting a low production of glucose and amino acids. Our transcriptomic analyses did show that glycogen biosynthesis was downregulated in symbionts under thermal stress. Therefore, the overall pattern of low expression of *Tret1*, *SLC7A1*, and *SLC7A2* may correspond to the reduced availability of glucose and amino acids during thermal stress. This low expression of transporter protein genes may simply reflect the dynamic interaction and coordination between coral host and symbionts under stress.

### Metabolic Integration of Coral and Symbiodiniaceae

**FIGURE 6:**
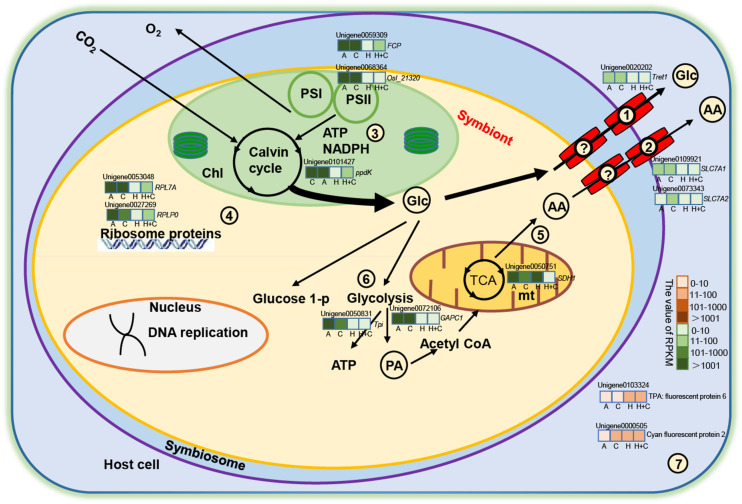
Metabolic integration of coral and Symbiodiniaceae. Only metabolic processes and several genes considered directly related to the study are shown. The question mark indicates that no DEGs for transporter were detected in the symbiont. Glucose transporter from symbiont (?), amino acid transporter from symbiont (?), glucose transporter from coral **(1)**, and amino acid transporter from coral **(2)**. **(3)** Photosynthesis and the Calvin cycle. **(4)** Ribosomal protein. **(5)** The TCA cycle. **(6)** Glycolysis. **(7)** Fluorescent protein. Red and green color scale represents upregulation and downregulation, respectively. AA, amino acid; Chl, chloroplast; Glc, glucose; mt, mitochondria; PA, pyruvate; TCA, tricarboxylic acid cycle. Italic letters around unigenes are annotation genes (i.e., RPL7A), and Arial letters are annotation proteins (i.e., cyan fluorescent protein). Unigenes in the schematic are selected by key genes, differential expression (up/down), and the value of RPKM (widely varied). The color variations in green (down expression) and orange (up expression) bar mean that the values of RPKM range from 0 to 10, 11 to 100, 101 to 1,000, and >1,000. Each gene box is equally divided into four pieces (A, C, H, and H+C), sequentially representing ATAC, ATHC, HTAC, and HTHC treatment, respectively.

Our results provide molecular evidence that the number of symbionts in coral host is low under heat stress and, thus, support a model of metabolic integration, such as the one presented in Figure 6. In brief, the fact that symbionts cannot successfully establish symbiosis with coral host under high temperature may result from downregulation of several genes of ribosomal and DNA replication proteins in the symbionts, leading to low algal density. Meanwhile, heat stress negatively affects some vital enzymes or proteins in metabolic processes such as TCA, Calvin cycle, glycolysis, and photosynthesis. Due to the low algal population, the amount of fixed organic carbon obtained by the coral host is low. As a result, the genes related to glucose and amino acid transporters were downregulated. We hypothesize that these changes in symbiont metabolism would, in turn, result in fewer inorganic nutrients for the symbionts. In summary, the physiological and transcriptomic data demonstrated that ocean warming might be more deleterious to juvenile coral than ocean acidification.

## Data Availability Statement

The datasets generated for this study can be found in the all raw data from this study are available in the NCBI under the accession number SRR11802619-SRR11802621 and PRJNA633301.

## Author Contributions

YS, LJ, and HH conceived and designed the study. YS, LJ, SG, and XY conducted the experiments and performed the laboratory and transcriptomic analyses. YS analyzed the data and drafted the manuscript. LJ, SG, XL, YZ, MG, XY, GZ, JL, PQ, and HH contributed to the laboratory analyses and interpretation of the results. All authors contributed to the article and approved the submitted version.

## Conflict of Interest

The authors declare that the research was conducted in the absence of any commercial or financial relationships that could be construed as a potential conflict of interest.

## Supplementary Material

Supplementary Figure 1 Number of differentially expressed genes of *Acropora intermedia* and symbionts in ATHC (28°C and *p*CO_2_: ∼1200 μatm), HTAC (30.5°C and *p*CO_2_: ∼600 μatm), HTHC (30.5°C and *p*CO_2_: ∼1200 μatm) treatments. **(A)** represents coral host, and **(B)** represents symbionts. Unigenes were functionally categorized based on database searches and literature.

Supplementary Figure 2 Number of unigenes of differential expression genes function of the coral host and symbiont transcript in the ATHC (28°C and *p*CO_2_: ∼1200 μatm), HTAC (30.5°C and *p*CO_2_: ∼600 μatm), and HTHC (30.5°C and *p*CO_2_: ∼1200 μatm) treatments. **(A)** represents coral host, and **(B)** represents symbionts. Unigenes were functionally categorized based on database searches and literature.

Supplementary Figure 3 Heatmap of annotated unigenes related to protein synthesis in the symbionts in the ATAC (28°C and *p*CO_2_: ∼600 μatm), ATHC (28°C and *p*CO_2_: ∼1200 μatm), HTAC (30.5°C and *p*CO_2_: ∼600 μatm) and HTHC (30.5°C and *p*CO_2_: ∼1200 μatm) treatments. The heatmap was drawn based on log-transformed RPKM value of unigenes. The color bar in heatmap represents the variation of the value of RPKM (from white to blue means the expression value of unigene from 0 to 0.4). High expression (blue color) represents the high value of RPKM, which means the expression level of unigene among different groups or different unigenes is high. Unigenes were functionally categorized based on database searches and literature.

Supplementary Table 1 Summary of sequencing analysis of 12 samples from ATHC (28°C and *p*CO_2_: ∼1200 μatm), HTAC (30.5°C and *p*CO_2_: ∼600 μatm), and HTHC (30.5°C and *p*CO_2_: ∼1200 μatm) treatments.

Supplementary Table 2 List of genomic and transcriptomic resources downloaded and used to filter the *Acropora intermedia* holobiont transcriptome into coral and symbiont compartments.

Supplementary Table 3 Statistical results of generalized linear models and *post-hoc* comparisons (*P*-values) examining the effects of temperature (T) and *p*CO_2_ on the survival, budding, symbiosis establishment, and green fluorescence of *Acropora intermedia*.

Supplementary Table 4 Transcriptome statistics for coral-special, symbiont, and coral-symbiont.

## References

[B1] Abrego D. Ulstrup K. E. Willis B. L. Van Oppen M. J. (2008). Species–specific interactions between algal endosymbionts and coral hosts define their bleaching response to heat and light stress. *Proc. R. Soc. B.* 275 2273–2282. 10.1098/rspb.2008.0180 18577506 PMC2603234

[B2] Abrego D. Van Oppen M. J. Willis B. L. (2009). Highly infectious symbiont dominates initial uptake in coral juveniles. *Mol. Ecol.* 18 3518–3531. 10.1111/j.1365-294X.2009.04275.x 19627495

[B3] Abrego D. Willis B. L. Van Oppen M. J. (2012). Impact of light and temperature on the uptake of algal symbionts by coral juveniles. *PLoS One* 7:e50311. 10.1371/journal.pone.0050311 23185603 PMC3504000

[B4] Ali A. Kriefall N. G. Emery L. E. Kenkel C. D. Matz M. V. Davies S. W. (2019). Recruit symbiosis establishment and *Symbiodiniaceae* composition influenced by adult corals and reef sediment. *Coral Reefs* 38 405–415. 10.1007/s00338-019-01790-z

[B5] Anthony K. Kline D. Diaz-Pulido G. Dove S. Hoegh-Guldberg O. (2008). Ocean acidification causes bleaching and productivity loss in coral reef builders. *Proc. Natl. Acad. Sci. U.S.A.* 105:17442. 10.1073/pnas.0804478105 18988740 PMC2580748

[B6] Baird A. H. Guest J. R. Willis B. L. (2009). Systematic and biogeographical patterns in the reproductive biology of scleractinian corals. *Annu. Rev. Ecol. Evol. Syst.* 40 551–571. 10.1146/annurev.ecolsys.110308.120220

[B7] Bollati E. D’angelo C. Alderdice R. Pratchett M. Ziegler M. Wiedenmann J. (2020). Optical feedback loop involving dinoflagellate symbiont and scleractinian host drives colorful coral bleaching. *Curr. Biol.* 30 1–13. 10.1016/j.cub.2020.04.055 32442463

[B8] Byrne M. Przeslawski R. (2013). Multistressor impacts of warming and acidification of the ocean on marine invertebrates’ life histories. *Integr. Comp. Biol.* 53 582–596. 10.1093/icb/ict049 23697893

[B9] Cérec V. Piquet-Pellorce C. Aly H. A. Touzalin A.-M. Jégou B. Bauché F. (2007). Multiple pathways for cationic amino acid transport in rat seminiferous tubule cells. *Biol. Reprod.* 76 241–249. 10.1095/biolreprod.106.056168 17065601

[B10] Cziesielski M. J. Schmidt-Roach S. Aranda M. (2019). The past, present, and future of coral heat stress studies. *Ecol. Evol.* 9 10055–10066. 10.1002/ece3.5576 31534713 PMC6745681

[B11] Davies S. W. Ries J. B. Marchetti A. Castillo K. D. (2018). *Symbiodinium* functional diversity in the coral *Siderastrea siderea* is influenced by thermal stress and reef environment, but not ocean acidification. *Front. Mar. Sci.* 5:150 10.3389/fmars.2018.00150

[B12] Desalvo M. K. Voolstra C. R. Sunagawa S. Schwarz J. A. Stillman J. H. Coffroth M. A. (2008). Differential gene expression during thermal stress and bleaching in the Caribbean coral *Montastraea faveolata*. *Mol. Ecol.* 17 3952–3971. 10.1111/j.1365-294X.2008.03879.x 18662230

[B13] Fabricius K. E. (2005). Effects of terrestrial runoff on the ecology of corals and coral reefs: review and synthesis. *Mar. Pollut. Bull.* 50 125–146. 10.1016/j.marpolbul.2004.11.028 15737355

[B14] Fernie A. R. Carrari F. Sweetlove L. J. (2004). Respiratory metabolism: glycolysis, the TCA cycle and mitochondrial electron transport. *Curr. Opin. Plant Biol.* 7 254–261. 10.1016/j.pbi.2004.03.007 15134745

[B15] Ferrier M. D. (1994). *Fluxes and Metabolic Pools of Amino Acids in Algal-Cnidarian Symbioses.* Ph. D. thesis, University of Maryland, Maryland.

[B16] Foster T. Gilmour J. P. Chua C. M. Falter J. L. Mcculloch M. T. (2015). Effect of ocean warming and acidification on the early life stages of subtropical *Acropora spicifera*. *Coral Reefs* 34 1217–1226. 10.1007/s00338-015-1342-7

[B17] Gong S. Xiao Y. Xu L. (2018). Flexible symbiotic associations of *Symbiodinium* with five typical coral species in tropical and subtropical reef regions of the Northern South China Sea. *Front. Microbiol.* 9:2485. 10.3389/fmicb.2018.02485 30450084 PMC6225575

[B18] Grabherr M. G. Haas B. J. Moran Y. Levin J. Z. Thompson D. A. Ido A. (2011). Full-length transcriptome assembly from RNA-Seq data without a reference genome. *Nat. Biotechnol.* 29:644. 10.1038/nbt.1883 21572440 PMC3571712

[B19] Graham E. M. Baird A. H. Willis B. L. Connolly S. R. (2013). Effects of delayed settlement on post-settlement growth and survival of scleractinian coral larvae. *Oecologia* 173 431–438. 10.1007/s00442-013-2635-6 23525803

[B20] Gust K. A. Najar F. Z. Habib T. Lotufo G. R. Piggot A. M. Fouke B. W. (2014). Coral-zooxanthellae meta-transcriptomics reveals integrated response to pollutant stress. *BMC Genomics* 15:591. 10.1186/1471-2164-15-591 25016412 PMC4117956

[B21] Harrison P. L. (2011). “Sexual reproduction of scleractinian corals,” in *Coral Reefs: An Ecosystem in Transition*, eds Dubinsky Z. Stambler N. (Dordrecht: Springer), 59–85. 10.1007/978-94-007-0114-4_6

[B22] Heyward A. J. Negri A. P. (1999). Natural inducers for coral larval metamorphosis. *Coral Reefs* 18 273–279. 10.1007/s003380050193

[B23] Hillyer K. E. Dias D. A. Lutz A. Roessner U. Davy S. K. (2017a). Mapping carbon fate during bleaching in a model cnidarian symbiosis: the application of 13C metabolomics. *New Phytol.* 214 1551–1562. 10.1111/nph.14515 28272836

[B24] Hillyer K. E. Dias D. A. Lutz A. Wilkinson S. P. Roessner U. Davy S. K. (2017b). Metabolite profiling of symbiont and host during thermal stress and bleaching in the coral *Acropora aspera*. *Coral Reefs* 36 105–118. 10.1007/s00338-016-1508-y

[B25] Hughes T. P. Baird A. H. Bellwood D. R. Card M. Connolly S. R. Folke C. (2003). Climate change, human impacts, and the resilience of coral reefs. *Science* 301 929–933. 10.1126/science.1085046 12920289

[B26] Hughes T. P. Kerry J. T. Lvarez-Noriega M. Lvarez-Romero J. G. Anderson K. D. Baird A. H. (2017). Global warming and recurrent mass bleaching of corals. *Nature* 543:373. 10.1038/nature21707 28300113

[B27] Humanes A. Noonan S. H. C. Willis B. L. Fabricius K. E. Negri A. P. (2016). Cumulative effects of nutrient enrichment and elevated temperature compromise the early life history stages of the coral *Acropora tenuis*. *PLoS One* 11:e0161616. 10.1371/journal.pone.0161616 27575699 PMC5004850

[B28] IPCC (2007). *Climate Change 2007: The Physical Science Basis: Contribution of Working Group I to the Fourth Assessment Report of the International Panel on Climate Change.* New York, NY: Cambridge University Press.

[B29] Jiang L. Zhang F. Guo M. L. Guo Y. J. Zhang Y. Y. Zhou G. W. (2017). Increased temperature mitigates the effects of ocean acidification on the calcification of juvenile *Pocillopora damicornis*, but at a cost. *Coral Reefs* 37 71–79. 10.1007/s00338-017-1634-1

[B30] Jorgensen P. Tyers M. (2004). How cells coordinate growth and division. *Curr. Biol.* 14 1014–1027. 10.1016/j.cub.2004.11.027 15589139

[B31] Kenkel C. D. Moya A. Strahl J. Humphrey C. Bay L. K. (2017). Functional genomic analysis of corals from natural CO_2_-seeps reveals core molecular responses involved in acclimatization to ocean acidification. *Glob. Chang. Biol.* 24 158–171. 10.1111/gcb.13833 28727232

[B32] Lajeunesse T. Trench R. (2000). Biogeography of two species of *Symbiodinium* (Freudenthal) inhabiting the intertidal sea anemone *Anthopleura elegantissima* (Brandt). *Biol. Bull.* 199 126–134. 10.2307/1542872 11081711

[B33] Lajeunesse T. C. Parkinson J. E. Gabrielson P. W. Jeong H. J. Santos S. R. (2018). Systematic revision of *Symbiodiniaceae* highlights the antiquity and diversity of coral endosymbionts. *Curr. Biol.* 28 2570–2580. 10.1016/j.cub.2018.07.008 30100341

[B34] Lesser M. P. (1997). Oxidative stress causes coral bleaching during exposure to elevated temperatures. *Coral Reefs* 16 187–192. 10.1007/s003380050073

[B35] Lewis E. Wallace D. Allison L. J. (1998). *Program Developed for CO_2_ System Calculations.* Tennessee: US Department of Energy.

[B36] Mohamed A. R. Andrade N. Moya A. Chan C. X. Negri A. P. Bourne D. G. (2019). Transcriptomic insights into the establishment of coral-algal symbioses from the symbiont perspective. *bioRxiv* 652131 10.1101/652131

[B37] Mortazavi A. Williams B. A. Mccue K. Schaeffer L. Wold B. (2008). Mapping and quantifying mammalian transcriptomes by RNA-Seq. *Nat. Methods* 5:621. 10.1038/nmeth.1226 18516045 PMC13303166

[B38] Nitschke M. R. Davy S. K. Ward S. (2016). Horizontal transmission of *Symbiodinium* cells between adult and juvenile corals is aided by benthic sediment. *Coral Reefs* 35 335–344. 10.1007/s00338-015-1349-0

[B39] Palmer C. V. Modi C. K. Mydlarz L. D. (2009). Coral fluorescent proteins as antioxidants. *PLoS One* 4:e7298. 10.1371/journal.pone.0007298 19806218 PMC2752795

[B40] Pochon X. Montoya-Burgos J. Stadelmann B. Pawlowski J. (2006). Molecular phylogeny, evolutionary rates, and divergence timing of the symbiotic dinoflagellate genus *Symbiodinium*. *Mol. Phylogenet. Evol.* 38 20–30. 10.1016/j.ympev.2005.04.028 15978847

[B41] Polato N. R. Voolstra C. R. Schnetzer J. Desalvo M. K. Randall C. J. Szmant A. M. (2010). Location-Specific responses to thermal stress in larvae of the reef-building coral *Montastraea faveolata*. *PLoS One* 5:e011221. 10.1371/journal.pone.0011221 20585643 PMC2890407

[B42] Roth M. S. Deheyn D. D. (2013). Effects of cold stress and heat stress on coral fluorescence in reef-building corals. *Sci. Rep.* 3:1421. 10.1038/srep01421 23478289 PMC3594756

[B43] Schnitzler C. E. Hollingsworth L. L. Krupp D. A. Weis V. M. (2012). Elevated temperature impairs onset of symbiosis and reduces survivorship in larvae of the Hawaiian coral, *Fungia scutaria*. *Mar. Biol.* 159 633–642. 10.1007/s00227-011-1842-0

[B44] Schwarz J. A. Krupp D. A. Weis V. M. (1999). Late larval development and onset of symbiosis in the scleractinian coral *Fungia scutaria*. *Biol. Bull.* 196 70–79. 10.2307/1543169 25575388

[B45] Silverstein R. N. Ross C. Baker A. C. (2015). Change in algal symbiont communities after bleaching, not prior heat exposure, increases heat tolerance of reef corals. *Glob. Chang. Biol.* 21 236–249. 10.1111/gcb.12706 25099991

[B46] Suwa R. Nakamura M. Morita M. Shimada K. Iguchi A. Sakai K. (2010). Effects of acidified seawater on early life stages of scleractinian corals (Genus *Acropora*). *Fish. Sci.* 76 93–99. 10.1007/s12562-009-0189-7

[B47] Tong H. Cai L. Zhou G. Yuan T. Zhang W. Tian R. (2017). Temperature shapes coral-algal symbiosis in the South China Sea. *Sci. Rep.* 7 1–12. 10.1038/srep40118 28084322 PMC5234030

[B48] Van Oppen M. J. Lough J. M. (eds) (2018). “Synthesis: coral bleaching: patterns, processes, causes and consequences,” in *Coral Bleaching*, (Berlin: Springer). 10.1007/978-3-540-69775-6_1

[B49] Voolstra C. R. Schnetzer J. Peshkin L. Randall C. J. Szmant A. M. Medina M. (2009). Effects of temperature on gene expression in embryos of the coral *Montastraea faveolata*. *BMC Genom.* 10:627. 10.1186/1471-2164-10-627 20030803 PMC2807443

[B50] Warner M. E. Fitt W. K. Schmidt G. W. (1999). Damage to photosystem II in symbiotic dinoflagellates: a determinant of coral bleaching. *Proc. Natl. Acad. Sci. U.S.A.* 96 8007–8012. 10.1073/pnas.96.14.8007 10393938 PMC22178

[B51] Weis V. M. (2008). Cellular mechanisms of Cnidarian bleaching: stress causes the collapse of symbiosis. *J. Exp. Biol.* 211 3059–3066. 10.1242/jeb.009597 18805804

[B52] Yakovleva I. Baird A. Yamamoto H. Bhagooli R. Nonaka M. Hidaka M. (2009). Algal symbionts increase oxidative damage and death in coral larvae at high temperatures. *Mari. Ecol. Prog.* 378 105–112. 10.3354/meps07857

[B53] Yorifuji M. Harii S. Nakamura R. Fudo M. (2017). Shift of symbiont communities in *Acropora tenuis* juveniles under heat stress. *Peer J.* 5:e4055. 10.7717/peerj.4055 29255647 PMC5732543

[B54] Yuyama I. Harii S. Hidaka M. (2012). Algal symbiont type affects gene expression in juveniles of the coral *Acropora tenuis* exposed to thermal stress. *Mar. Environ. Res.* 76 41–47. 10.1016/j.marenvres.2011.09.004 22001189

[B55] Yuyama I. Ishikawa M. Nozawa M. Yoshida M.-A. Ikeo K. (2018). Transcriptomic changes with increasing algal symbiont reveal the detailed process underlying establishment of coral-algal symbiosis. *Sci. Rep.* 8 1–11. 10.1038/s41598-018-34575-5 30429501 PMC6235891

[B56] Zhou G. Cai L. Li Y. Tong H. Jiang L. Zhang Y. (2017). Temperature-Driven local acclimatization of *Symbiodnium* hosted by the coral *Galaxea fascicularis* at Hainan Island, China. *Front. Microbiol.* 8:2487. 10.3389/fmicb.2017.02487 29312196 PMC5733085

[B57] Zhou G. Huang H. Lian J. Zhang C. Li X. (2012). Habitat correlation of *Symbiodinium* diversity in two reef-building coral species in an upwelling region, eastern Hainan Island, China. *J. Mar. Biol. Assoc. U.K.* 92 1309–1316. 10.1017/S0025315411001548

