# Supplementary material for: Impact of Ocean Warming and Acidification on Symbiosis Establishment and Gene Expression Profiles in Recruits of Reef Coral Acropora intermedia
Source: Front Microbiol. 2020 Sep 30;11:532447. doi: 10.3389/fmicb.2020.532447 (PMC7561415; doi:10.3389/fmicb.2020.532447)
Supplement: Supplementary Figure 1 — Number of differentially expressed genes of Acropora intermedia and symbionts in ATHC (28°C and pCO2: ∼1200 μatm), HTAC (30.5°C and pCO2: ∼600 μatm), HTHC (30.5°C and pCO2: ∼1200 μatm) treatments. (A) represents coral host, and (B) represents symbionts. Unigenes were functionally categorized based on database searches and literature. [file Data_Sheet_1.docx]

**Supplementary Material**

**Supplementary Table 1.** Summary of sequencing analysis of 12 samples from ATHC (28℃ and pCO2: ~1200 µatm), HTAC (30.5℃ and pCO2: ~600 µatm), HTHC (30.5℃ and pCO2: ~1200 µatm) treatments.

| **Sample** | **Raw reads** | **Clean reads** | **Clean bases** | **Error(%)** | **Q20(%)** | **Q30(%)** | **GC(%)** |
| --- | --- | --- | --- | --- | --- | --- | --- |
| **A1** | 51,276,282 | 49,542,392 | 7.43G | 0.02 | 97.21 | 92.95 | 43.20 |
| **A2** | 43,582,558 | 42,115,750 | 6.32G | 0.02 | 97.01 | 92.52 | 42.69 |
| **A3** | 49,377,344 | 47,589,230 | 7.14G | 0.02 | 97.14 | 92.81 | 43.14 |
| **B1** | 48,650,202 | 46,960,210 | 7.05G | 0.02 | 96.90 | 92.30 | 42.62 |
| **B2** | 49,050,646 | 47,363,516 | 7.11G | 0.02 | 97.07 | 92.67 | 42.74 |
| **B3** | 48,182,704 | 46,563,612 | 6.99G | 0.02 | 97.09 | 92.74 | 42.30 |
| **C1** | 46,266,404 | 44,672,394 | 6.70G | 0.01 | 97.48 | 93.33 | 42.43 |
| **C2** | 51,485,160 | 49,688,828 | 7.46G | 0.02 | 97.39 | 93.13 | 42.36 |
| **C3** | 44,786,678 | 43,178,990 | 6.48G | 0.02 | 97.26 | 92.85 | 42.48 |
| **D1** | 47,721,028 | 46,064,856 | 6.91G | 0.01 | 97.58 | 93.5 | 42.18 |
| **D2** | 53,332,812 | 51,260,964 | 7.69G | 0.01 | 97.74 | 93.96 | 42.36 |
| **D3** | 56,215,636 | 54,136,998 | 8.12G | 0.01 | 97.81 | 94.14 | 42.78 |
| **Total** | 289,711,812 | 281,021,672 | 85.4G |  |  |  |  |

Note: A1, A2 and A3: sampling from ATAC treatment. B1, B2 and B3: sampling from ATHC treatment. C1, C2 and C3: sampling from HTAC treatment. D1, D2 and D3: sampling from HTHC treatment.

**Supplementary Table 2.** List of genomic and transcriptomic resources downloaded and used to filter the *Acropora intermedia* holobiont transcriptome into coral and symbiont compartments.

| **Taxon** | **Species** | **Download Source** |
| --- | --- | --- |
| anemone | *Nematostella vectensis* | https://www.ncbi.nlm.nih.gov/genome/?term=Nematostella+vectensis |
| coral | *Acropora palmata* | reefgenomics.org |
|  | *Acropora digitifera* | https://www.ncbi.nlm.nih.gov/genome/?term=Acropora+digitifera |
|  | *Acropora hyacinthus* | reefgenomics.org |
| **Taxon** | **Type** | **Download Source** |
| zooxanthellae | *Symbiodinium* (*Symbiodinium* *microadriaticum*) | https://www.ncbi.nlm.nih.gov/genome/?term=Symbiodinium+microadriaticum |
|  | *Fugacium* (*Symbiodinium* *kawagutii*) | http://web.malab.cn/symka_new/download.jsp |
|  | *Breviolum* (*Symbiodinium minutum*) | https://www.ncbi.nlm.nih.gov/genome/?term=Symbiodinium+minutum |
|  | *Symbiodinium* (*Symbiodinium* sp. clade A Y106) | https://www.ncbi.nlm.nih.gov/genome/?term=Symbiodinium+sp.+clade+A+Y106 |
|  | *Cladocopium* (*Symbiodinium* sp. clade C Y103) | https://www.ncbi.nlm.nih.gov/genome/?term=Symbiodinium+sp.+clade+C+Y103 |
|  | *Symbiodinium* (*clade A1*(culture)) | https://www.ncbi.nlm.nih.gov/Traces/wgs/?val=GAKY01 |
|  | *Symbiodinium* (*Clade A2* (culture)) | https://www.ncbi.nlm.nih.gov/Traces/wgs/?val=GBGW01 |
|  | *Breviolum* (*Clade B2* (culture)) | https://www.ncbi.nlm.nih.gov/Traces/wgs/?val=GBRZ01 |
|  | *Cladocopium* (*Clade C* (culture)) | <https://www.ncbi.nlm.nih.gov/Traces/wgs/?val=GBSC01>  https://www.ncbi.nlm.nih.gov/Traces/wgs/?val=GAFO01 |
|  | *Durusdinium* (Clade D (culture)) | <https://www.ncbi.nlm.nih.gov/Traces/wgs/?val=GBRR01>  https://www.ncbi.nlm.nih.gov/Traces/wgs/?val=GAFP01 |
|  | *Symbiodinium* (*Symbiodinium* KB8 (clade A)) | http://medinalab.org/zoox/ |
|  | *Breviolum* (Mf1.05b (clade B)) | http://medinalab.org/zoox/ |

**Supplementary Table 3.** Statistical results of generalized linear models and post-hoc comparisons (*P*-values) examining the effects of temperature (T) and *p*CO_2_ on the survival, budding, symbiosis establishment, and green fluorescence of *Acropora intermedia*.

| EFFECT | SS | *df* | MS | *F* | *P* |
| --- | --- | --- | --- | --- | --- |
| Survivorship |  |  |  |  |  |
| *T* | 0.010 | 1 | 0.010 | 0.800 | 0.379 |
| *p*CO_2_ | 0.004 | 1 | 0.004 | 0.329 | 0.571 |
| *T* 🞨 *p*CO_2_ | 0.0002 | 1 | 0.0002 | 0.015 | 0.902 |
| Error | 0.366 | 28 | 0.013 |  |  |
| Symbiont infection |  |  |  |  |  |
| *T* | 4.378 | 1 | 4.378 | 248.110 | **＜0.001** |
| *p*CO_2_ | 0.001 | 1 | 0.001 | 0.049 | 0.827 |
| *T* 🞨 *p*CO_2_ | 0.001 | 1 | 0.001 | 0.049 | 0.827 |
| Error | 0.494 | 28 | 0.018 |  |  |
| Budding |  |  |  |  |  |
| *T* | 0.062 | 1 | 0.062 | 8.868 | **0.006** |
| *p*CO_2_ | 0.018 | 1 | 0.018 | 2.533 | 0.123 |
| *T* 🞨 *p*CO_2_ | 0.018 | 1 | 0.018 | 2.533 | 0.123 |
| Error | 0.195 | 28 | 0.007 |  |  |
| Green fluorescent |  |  |  |  |  |
| T | 1.848 | 1 | 1.848 | 217.715 | **＜0.001** |
| *p*CO_2_ | 0.022 | 1 | 0.022 | 2.636 | 0.116 |
| *T* 🞨 *p*CO_2_ | 0.003 | 1 | 0.003 | 0.381 | 0.542 |
| Error | 0.238 | 28 |  |  |  |

**Supplementary Table 4**. Transcriptome statistics for coral-special, symbiont, and coral-symbiont.

|  | Gene number | GC % | N50 | Max length | Min length | Average length | Total assembled |
| --- | --- | --- | --- | --- | --- | --- | --- |
| Coral-special | 49,107 | 39.90 | 1,085 | 15,928 | 201 | 697 | 34,240,695 |
| Symbiont-special | 2741 | 57.11 | 472 | 2,574 | 201 | 434 | 1,191,742 |
| Coral-symbiont | 15 | 52.52 | 334 | 586 | 225 | 332 | 4,981 |

**Supplementary Figure 1**. Number of differentially expressed genes of *Acropora intermedia* and symbionts in ATHC (28℃ and *p*CO_2_: ~1200 µatm), HTAC (30.5℃ and *p*CO_2_: ~600 µatm), HTHC (30.5℃ and *p*CO_2_: ~1200 µatm) treatments. A represents coral host, and B represents symbionts.

 **Supplementary Figure 2:** Number of unigenes of differential expression genes function of the coral host and symbiont transcript in the ATHC (28℃ and *p*CO_2_: ~1200 µatm), HTAC (30.5℃ and *p*CO_2_: ~600 µatm), and HTHC (30.5℃ and *p*CO_2_: ~1200 µatm) treatments. A represents coral host, and B represents symbionts. The functional categories of unigenes are designated by database searches and literature.

**
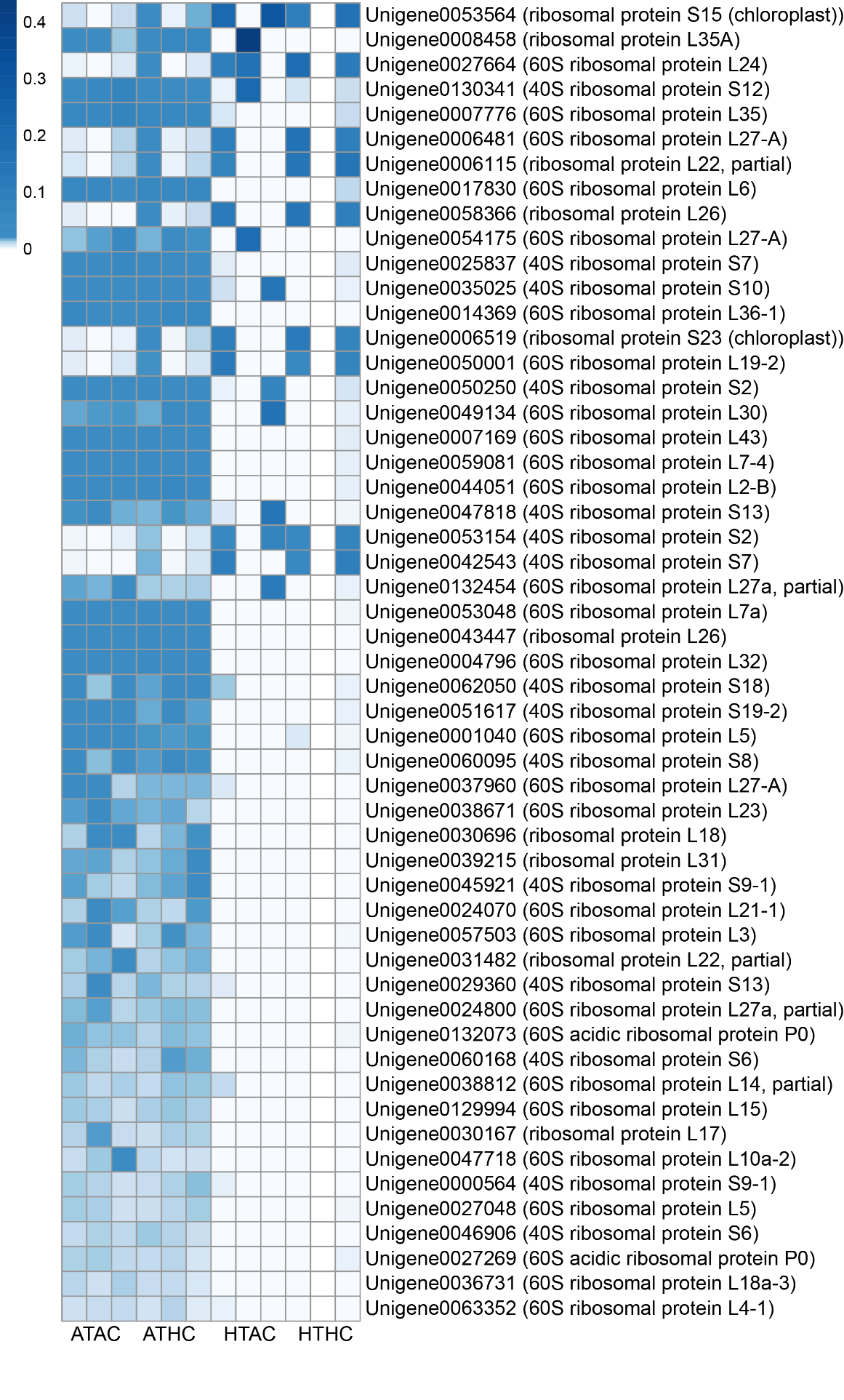
**

**Supplementary Figure 3:** Heatmap of annotated unigenes related to protein synthesis in the symbionts in the ATAC (28℃ and *p*CO_2_: ~600 µatm ), ATHC (28℃ and *p*CO_2_: ~1200 µatm), HTAC (30.5℃ and *p*CO_2_: ~600 µatm) and HTHC (30.5℃ and *p*CO_2_: ~1200 µatm) treatments. The heatmap was drawn based on log-transformed RPKM value of unigenes. The color bar in heatmap represents the variation of the value of RPKM (from white to blue means the expression value of unigene from 0 to 0.4). High expression (blue color) represents the high value of RPKM, which means the expression level of unigene among different groups or different unigenes is high. Unigenes were functionally categorized based on database searches and literature.
